# An Engineered Non-Toxic Superantigen Increases Cross Presentation of Hepatitis B Virus Nucleocapsids by Human Dendritic Cells

**DOI:** 10.1371/journal.pone.0093598

**Published:** 2014-04-01

**Authors:** Julie D. McIntosh, Kristy Manning, Shilpa Chokshi, Nikolai V. Naoumov, John D. Fraser, P. Rod Dunbar, John A. Taylor

**Affiliations:** 1 School of Biological Sciences and Maurice Wilkins Centre for Molecular Biodiscovery, University of Auckland, Auckland, New Zealand; 2 School of Medical Sciences and Maurice Wilkins Centre for Molecular Biodiscovery, University of Auckland, Auckland, New Zealand; 3 Institute of Hepatology, University College London, London, United Kingdom; University of Melbourne, Australia

## Abstract

Virus like particles (VLPs) are potent immunogens capable of priming strong protective antibody responses due to their repetitive structural arrangement and affinity for specific B cell receptors. By contrast, T cell responses to VLPs can be weak due to inefficient uptake and processing by antigen presenting cells. We report here a novel strategy for increasing the T cell reactivity of a VLP, the nucleocapsid of hepatitis B virus, through covalent coupling of M1, an engineered form of the Streptococcal superantigen SMEZ2, that binds MHC II with high affinity but lacks its T cell mitogenic capability. M1:HBcAg conjugates bound to dendritic cells and were efficiently endocytosed into late endosomes. Human dendritic cells pulsed with M1:HBcAgs stimulated HBV-specific CD8^+^ T cells more effectively than cells pulsed with native capsids indicating that the modified VLP was more effectively cross presented by APCs. Coupling of M1 was also able to induce significantly greater reactivity of human CD4^+^ T cells specific for a common T-helper epitope. These studies indicate the potential of recombinant superantigens to act as flexible molecular adjuvants that can be incorporated into various subunit vaccine platforms leading to enhanced T cell reactivity in humans.

## Introduction

Generation of cytotoxic T lymphocytes (CTL) by subunit vaccines requires cross-presentation of antigen to Class I Major Histocompatability Complex (MHC I) on the surface of antigen presenting cells (APCs) and cross-priming of antigen-specific CD8^+^ T cells. Dendritic cells, a specialized subset of APC represent critical targets of subunit vaccines due to their intrinsic ability to capture exogenous antigens and direct them into the cross presentation pathway. Cross presentation of subunit vaccines in mice can be enhanced by strategies designed to target antigens to specific receptors on the surface of DCs leading to receptor-mediated endocytosis of antigen and delivery to intracellular compartments where processing and cross presentation of key epitopes to MHC I occurs. For example, targeting antigen of the model antigen ovalbumin (OVA) to the lectin DEC-205 expressed on DCs through coupling of OVA to Dec-205-specific antibodies can significantly increase the efficiency of antigen presentation on MHC class I and II molecules leading to elevated numbers of CD4^+^ T and CD8^+^ T cells in vaccinated mice [1]–[3].

An alternative approach to targeting proteins to DCs utilises synthetic or natural ligands of DC surface proteins. For example, the family of calcium-dependent lectins (CLRs) has received significant attention as DC receptors to which antigens can be targeted after coupling to either antibody or a specific sugar moiety and several studies have reported significantly improved immune responses in both mouse models and human clinical trials (reviewed in [4]). A further extension of this approach is to utilise the specificity and affinity of natural protein ligands that are recognised by molecules present at the surface of the cell. Cell surface receptors that have been targeted with their natural ligands include heat-shock proteins [5], [6], bacterial-derived toxins [7] and C-type lectins [8].

Increasing the efficiency of cross presentation requires not only targeting to the DC surface but the subsequent delivery of antigen to an endocytic pathway that precedes processing and loading of epitopes to class I MHC. Different mechanisms for cross-presentation have been proposed largely based on studies with murine DC subsets. The ‘cytosolic pathway’ requires the transfer of internalized antigens to the cytosol where they are degraded by the proteasome. The resulting peptides are translocated into the endoplasmic reticulum (ER) by TAP transporters and loaded onto MHC class I molecules effectively entering the endogenous pathway. This model is supported by the observation that elements of the ER retrotranslocation machinery can be detected within specialised phagolysosomal compartments containing exogenous antigen [9]–[13]. Alternatively some antigens may be processed by endosomal proteases, MHC I loading being independent of TAP (reviewed in [14]). The precise mechanism(s) by which cross presentation occurs is likely to be dependant on the route of internalisation and the specific subset of DCs in which it occurs.

In this study we have explored a novel method for targeting a VLP to human monocyte derived DCs with the aim of improving the T cell responses to prospective VLP-based vaccines in humans. Our approach utilises conjugation to a bacterial superantigen engineered to lack its T cell mitogenic activity while retaining high affinity binding to MHC II on the surface of APCs. Internalization of VLPs via MHC II directs the particles to a late endosomal/lysosomal compartment where transfer of epitopes to MHC I can occur leading to cross priming of CD8^+^ T cells.

## Methods

### Ethics Statement

Blood was collected from healthy donors after obtaining written informed consent. All procedures related to blood collection were approved by The University of Auckland Human Subjects Ethics Committee.

### Expression and purification of HBcAg and SMEZ2.M1

cDNA encoding amino acids 1–142 of HBcAg was amplified by PCR from the viral genome (subtype *ayw*) and cloned into the plasmid pET17xb (Novagen). To facilitate covalent coupling using the bifunctional crosslinker sulpho-MBS, the wild-type HBcAg sequence was mutated to replace the proline and alanine at residues 79–80 respectively with the amino acid sequence GAKGG. In addition, the cysteine residues at positions 48 and 107 were mutated to alanine to ensure coupling of the M1 only occurred via the introduced cysteine as described previously [15]. Expression of HBcAg in E.coli was induced by 2 mM IPTG and the protein purified as described previously [16], [17]. Briefly, induced cells were lysed in 50 mM Tris HCl pH 7.4, 1 mM EDTA, 5 mM DTT, 1 mM Pefabloc, 0.1 mg/mL RNase and 0.1 mg/mL DNase and insoluble debris removed. Proteins was precipitated by addition of ammonium sulphate (40% saturation) at 4°C for 1 hour and resuspended in 100 mM Tris HCl pH 7.5, 150 mM NaCl, 50 mM sucrose 2 mM DTT. After dialysis to remove ammonium sulphate the sample was applied to a Sepharose 6 16/60 column (GE Healthcare Life Sciences, USA). The partially purified HBcAg was dissociated into dimers in 3.5 M urea at pH 9.5 and the protein further purified by a second gel filtration step using a Superdex 75 column (GE Healthcare Life Sciences, USA) equilibrated in 100 mM NaHCO_3_ pH 9.5, 2 mM DTT. The dimers were reformed into virus-like particles by addition of 0.2 volumes of Capsid Formation Buffer (0.5 mM HEPES pH 5.0, 2 M NaCl, 300 mM MgCl2, 10 mM DTT) and incubation at 21°C for 1 hour. HBcAg VLP were separated from protein aggregates by gel filtration using the Sepharose 6 16/60 column equilibrated in PBS. Purified protein was assessed by SDS-PAGE and the protein concentration was measured using the Pierce™ BCA protein assay reagent (Thermo Scientific, Rockford, IL.). Endotoxin was removed from recombinant protein preparations by Triton X-114 separation [18]. The resulting protein solution was tested for presence of endotoxin by LAL Gel Clot assay (Associates of Cape Cod Inc., East Falmouth, MA) according to the manufacturer's protocol. The expression and purification of SMEZ2.M1 from Streptoccoccus pyogenes has been described previously (hereafter referred to as M1) [19], [20].

### Coupling of HBcAg and TTp2 to M1

M1 was coupled to HBcAg using the heterobifunctional crosslinker maleimidobenzoic acid sulfosuccinimidyl ester (Sulfo-MBS, Thermo Scientific, Rockford, IL). Sulphydryl groups present on M1 were reduced by addition of 10 mM DTT followed by removal of the reducing agent with a ZEBA desalting column (Thermo Scientific, Rockford, IL). HBcAg was incubated with a 50-fold molar excess of sulfoMBS for 30 minutes at room temperature in PBS pH 7.4 and excess sulfoMBS was removed by desalting. HBcAg-sulfoMBS was incubated with M1 in PBS pH 7.8 overnight at 4°C. The coupled product was purified from unbound M1 by ultracentrifugation at 150,000×g for one hour and the M1:HBcAg was resuspended in PBS.

For coupling to TTp2 peptide, M1 was treated with 10 mM DTT and dialysed overnight at 4°C against PBS. For coupling, M1 (at a final concentration of 1 mg/ml) was incubated with a 10-fold molar excess of peptide in the presence of 200 mM Tris pH 8.0 and 50 µM CuSO_4_ (final concentration). The coupling reaction was performed at room temperature for 4 hours or overnight. Excess peptide was removed by buffer exchange through a Vivaspin protein concentrator (GE Healthcare Life Sciences, USA) with a 5 kDa cutoff followed by gel filtration chromatography using the Superdex 75 column equilibrated in PBS (pH 8.0). The coupled M1 was analysed by SDS-PAGE under non-reducing conditions and stained with Coomassie blue stain. The coupled products were filtered through a 0.22 micron filter prior to use.

### Cell Culture

Blood was collected from healthy donors under informed consent approved by an institutional human subjects ethics committee. PBMCs were isolated from heparinised blood by density gradient centrifugation using Lymphoprep™ (StemCell Technologies™, VIC, Australia). Monocytes were purified from PBMC using the Monocyte Isolation Kit II (MACS Miltenyi Biotec, USA) according to the manufacturer's instructions. Purity of the monocyte fraction was analysed by flow cytometry and determined to be >80%. Following purification, monocytes were cultured in RPMI/10% FCS containing GM-CSF (100 ng/ml, Peprotech) and IL-4 (50 ng/ml, Peprotech) and the cytokines replenished at two day intervals. Cells were harvested on day 6 as immature DCs (imDCs). If maturation of the DCs (mDCs) was required, 5 µg/ml LPS (Sigma Aldrich, New Zealand) was added to the culture medium for 24–48 hours. HLA*A2 status was determined by typing the blood with an anti-HLA*A2 antibody (AbD Serotec, BioRad, Auckland, New Zealand).

### Analysis of binding of M1:HBcAg, HBcAg or M1 to cell lines

DCs, prepared as described or the EBV-transformed B cell line LG-2 [21], (1×10^5^ cells) were incubated with the indicated amount of M1 or the HBcAg preparations in a final volume of 100 µl for 1 hour at 4°C. Nonbound HBcAg/M1 was removed by three washes of PBS at 4°C. Bound M1:HBcAg was detected by incubation with a rabbit anti-HBcAg antibody (DAKO, Denmark) and M1 was detected by incubation with rabbit anti-M1 serum (a kind gift from J.D. Fraser) at a 1/1000 dilution for 1 hour at 4°C. The cells were washed three times with PBS and subsequently incubated with ALEXA-Fluor-488 anti-rabbit IgG (Molecular Probes) for 30 minutes at 4C. Analysis of the surface bound florescence was performed by flow cytometry using a FACS calibre (BD Biosciences, New Zealand).

### Analysis of clonal T cell activation

The HBc_18–27_ cell line was derived from an acutely-infected HBV patient described previously [22] and was a kind gift from Professor Antonio Bertoletti. Activation of cytotoxic action of CD8^+^ cells was measured by simultaneously detecting CD107a expression at the cell surface and IFNγ secretion by intracellular staining. DCs from HLA*A2^+^ donors were replated in RPMI in a 96-well plate at 5×10^4^ cells/well. The DCs were incubated with antigens at the appropriate concentration for 60 minutes at room temperature. Following antigen loading, the cells were washed, resuspended in RPMI/10% FCS containing GM-CSF and IL-4 and incubated overnight to allow processing of antigen. The following day, HBc_18–27_ clone cells were added at a ratio of 1∶2 target to effector ratio. PE-labelled mouse anti-human CD107a (BD Biosciences, New Zealand) was added and the cells incubated at 37°C, 5% CO_2_ for 1 hour. BFA (5 µg/ml) and monensin (5 µg/ml) were added to the cells and the samples incubated for a further 5 hours at 37°C. Following the incubation, the cells were stained for CD8 to ensure only the HBc_18–27_ clone cells were assessed. Intracellular staining of IFNγ expression was done using the CytoFix/CytoPermTM Fixation/Permeabilisation Solution Kit (BD Biosciences, New Zealand) according to manufacturer's instructions. Briefly, the cells were incubated with BD Fixation/Permeabilisation solution (BD Biosciences, New Zealand) for 20 minutes on ice and the cells washed twice with BDPerm/WashTM (BD Biosciences, New Zealand). IFNγ gamma was detected with FITC-anti-human IFNγ (BD Biosciences, New Zealand) for 30 minutes on ice followed by two washes. The cells were analysed by flow cytometry using a FACS calibre (BD Biosciences, New Zealand).

### IFNγ ELISPOT Assay with PBMCs

The IFN-γ ELISPOT Assay (BD Biosciences, New Zealand) was performed using an indirect method [23], [24]. PBMCs (2×10^5^ cells/well in sRPMI/10% human serum) were cultured in the presence of the appropriate antigens or controls for 20 hours at 37°C. In all experiments duplicate or triplicate wells were used for each antigen. Monoclonal unlabelled anti-IFNγ capture antibody (100 µl of 5 µg/ml in sterile PBS) was incubated on nitrocellulose-bottom plates overnight at 4°C. Unbound capture antibody was removed and the wells washed with 200 µl sRPMI/10% human serum. The wells were subsequently incubated with 200 µl sRPMI/10% human serum for 2 hours at room temperature to block non-specific binding sites. Following the incubation, the blocking solution was removed and the cultures containing the PBMCs/antigens transferred to the antibody-coated wells. The plate was incubated for a further 24 hours at 37°C. The medium containing the cells/antigens was subsequently removed and the ELISPOT developed according to manufacturer's instructions. Cytokine spots were visualised with AEC substrate (3-Amino-9-Ethyl-carbazole, supplied by Sigma) and counted using an Elispot Plate reader (AID Elispot, Germany). Previous studies have indicated substantial diversity in terms of precursor frequency of Tetanus-specific cells within a population of individuals [25], [26] Therefore, initial screening ELISPOT assays were used to determine whether the individual had a detectable response to the tetanus toxoid protein. Only individuals with a response of greater than 100 IFNγ-producing cells/10^6^ PBMC (after correction for activation in the absence of stimulation) were used for further analysis (data not shown).

### CD4^+^ cell Depletion ELISPOT Assay

The CD4^+^ depletion assay was performed according to a method developed previously [27]. Briefly, CD4^+^ cells were separated from the PBMCs using positive selection with the CD4^+^ Microbeads (MACS Miltenyi Biotech) according to manufacturer's instructions. The purity of CD4^+^ cells in the positive fraction was shown to be >94% and the presence of CD4^+^ cells in the PBMC (CD4^−^) fraction was shown to be <6% according to flow cytometry analysis using anti-human CD4^+^ antibody. Following purification, 1×10^5^ CD4^+^ cells were added to 2×10^5^ irradiated (3000rad or 30Gy) PBMCS per well. For analysis of the CD4^−^ fraction 2×10^5^ cells/well PBMC from the CD4^+^ purification were analysed. The appropriate antigen was added to the well and the ELISPOT was continued in the indirect method as described.

## Results

### Covalent attachment of M1 to Hepatitis B virus core antigen (HBcAg) VLPs

SMEZ2 from *Streptococcus pyogenes* binds MHC II on the surface of specialised antigen presenting cells with high affinity (K_d_∼10^−9^ M). Utilizing the high resolution X-ray structure of SMEZ2 as a template [28], selective mutation of residues involved in binding the TCR resulted in the generation of SMEZ2.M1, (hereafter referred to as M1), a non toxic high-affinity ligand for MHC II [19], [20]. We sought to determine whether M1 could target a VLP to human DCs resulting in increased VLP-specific T cell reactivity.

**Figure 1:**
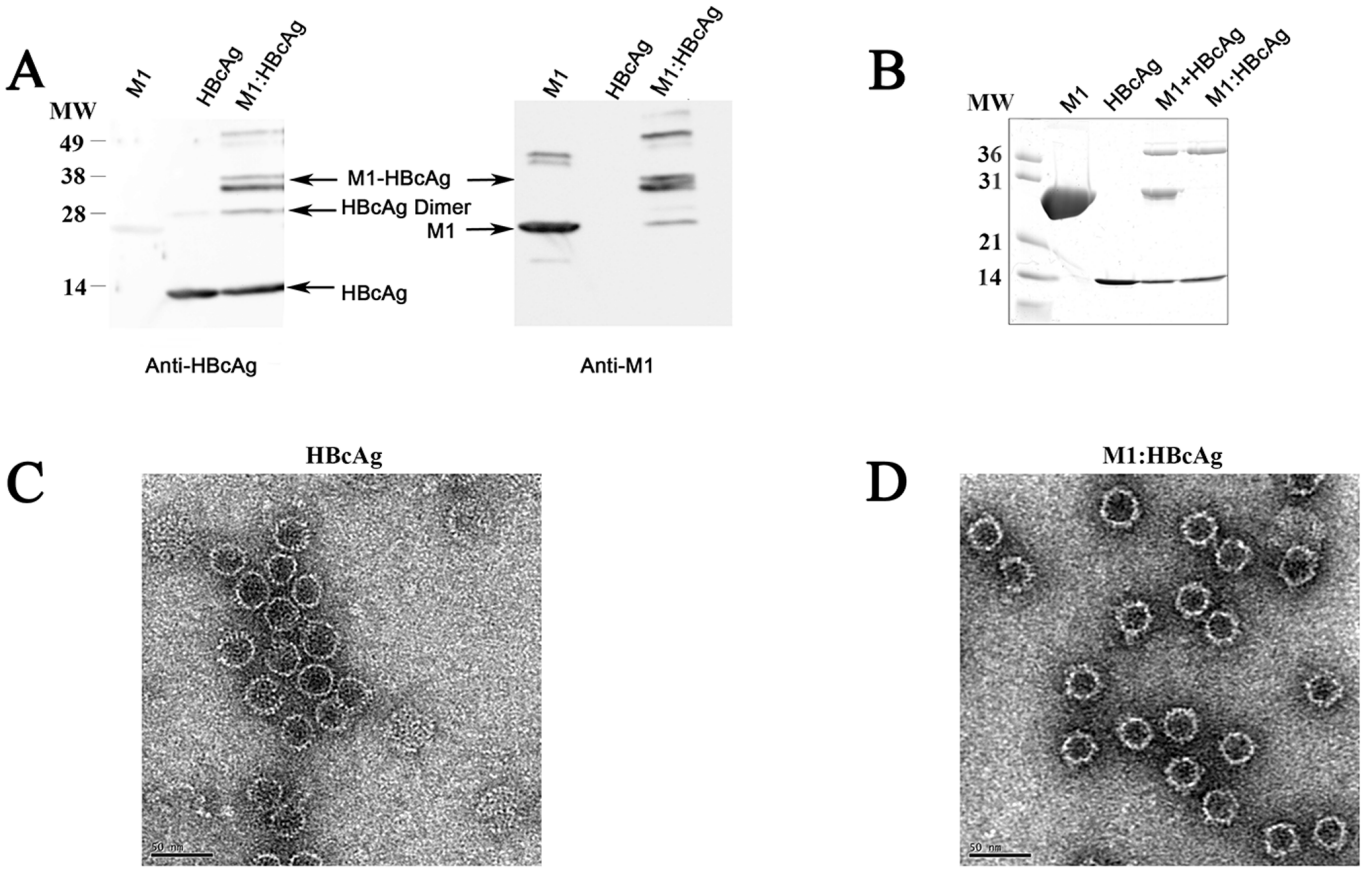
Synthesis and purification of M1:HBcAg VLPs. M1 was coupled to HBcAg (residues 1–142) using the heterobifunctional cross-linker sMBS (Pierce). Western Blot indicating the coupled product is recognised by both the anti-M1 and anti-HBcAg antibodies. (B) Following conjugation, the M1:HBcAg was purified from unbound M1 protein by ultracentrifugation. The resulting coupled product was analysed by SDS-PAGE under reducing conditions followed by silver stain. Following purification no contaminating M1 was observed in the purified product. HBcAg (C) and M1:HBcAg (D) were stained with uranyl acetate and analysed by electron microscopy. The morphology of HBcAg was retained following coupling and purification.

VLPs were produced by recombinant expression of residues 1–142 of hepatitis B virus core antigen (HBcAg) in *E.coli*. Particles were purified by gel filtration chromatography [16], [17] and coupled to M1 using the heterobifunctional cross-linker sulfo MBS (sMBS) [15]. The resulting complex, hereafter refered to as M1:HBcAg, migrated as a doublet at the expected size by SDS PAGE (∼39 kDa); both bands in the doublet were detected by immunoblotting with antibodies to both M1 and HBcAg (Figure 1A). Unbound M1 was separated from M1:HBcAg by ultracentrifugation and the purity of the coupled product was confirmed by silver staining (Figure 1B). The relative efficiency of coupling was determined by denisitometric analysis of SDS PAGE gels after staining with Sypro Ruby and revealed approximately 12 molecules of M1 per VLP, a coupling efficiency of ∼5% (data not shown). Electron microscopy confirmed the ability of the mutated sequence to form VLPs that were indistinguishable from wild-type capsids and showed that the M1:HBcAg retained this morphology (Figure 1C and D).

### M1:HBcAg bind to cells expressing MHC II

**Figure 2:**
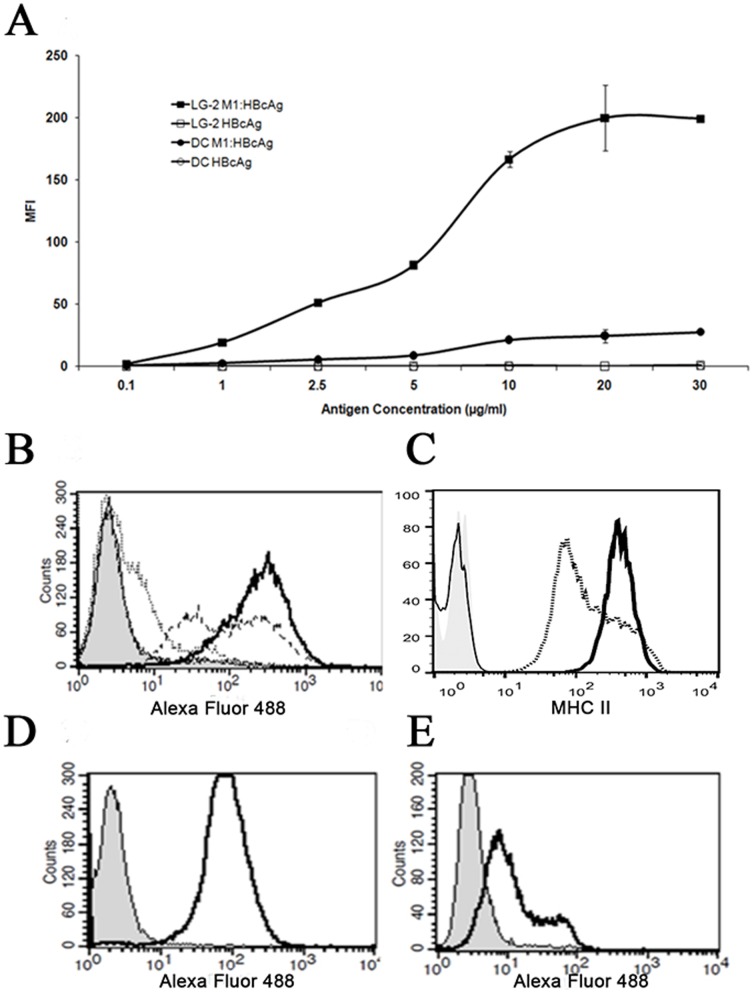
Dose-dependent binding of M1:HBcAg to immature DCs and B cells. A, immature DCs and LG-2 cells were incubated with increasing amounts of M1:HBcAg or HBcAg on ice. Following extensive washing, bound HBcAg was detected using a rabbit anti-HBcAg antibody followed by anti-rabbit IgG AlexaFluor-488. B, Dose-dependent binding of M1:HBcAg on LG-2 cells. Cells were incubated with 1 µg/ml (thin line), 5 µg/ml (dashed line) or 30 µg/ml (solid line) M1:HBcAg. Cells stained with secondary antibody alone are shown in shaded grey. C, Levels of MHC II on the surface of LG-2 (solid line) and DCs (dashed line) were compared by flow cytometry. Binding of M1 (50 µg/ml) to LG-2 cells (D) and DCs (E) is revealed by a shift in fluorescence after staining with anti-M1 (shaded peak represents unstained cells).

To confirm that M1 retains its ability to bind to MHC II when covalently coupled to HBcAg we tested the ability of the VLPs to bind to human MoDCs and LG-2, a cell line derived from human B cells, both of which express MHC II on the cell surface. M1:HBcAg bound to these cells in a dose-dependent, saturable manner whereas unmodified HBcAg exhibited little or no binding at any of the concentrations tested (Figure 2A and B). The level of M1:HBcAgs bound to LG-2 cells was ∼10 fold greater than MoDCs. Flow cytometric analysis of unconjugated M1 bound to each cell type in excess concentrations confirmed that the capacity for M1 binding was similarly greater for LG-2 cells most likely due to increased MHC II expression at the plasma membrane (Figure 2C–E).

### M1 targets VLPs to an endosomal/lysosomal compartment that contains MHC I

**Figure 3:**
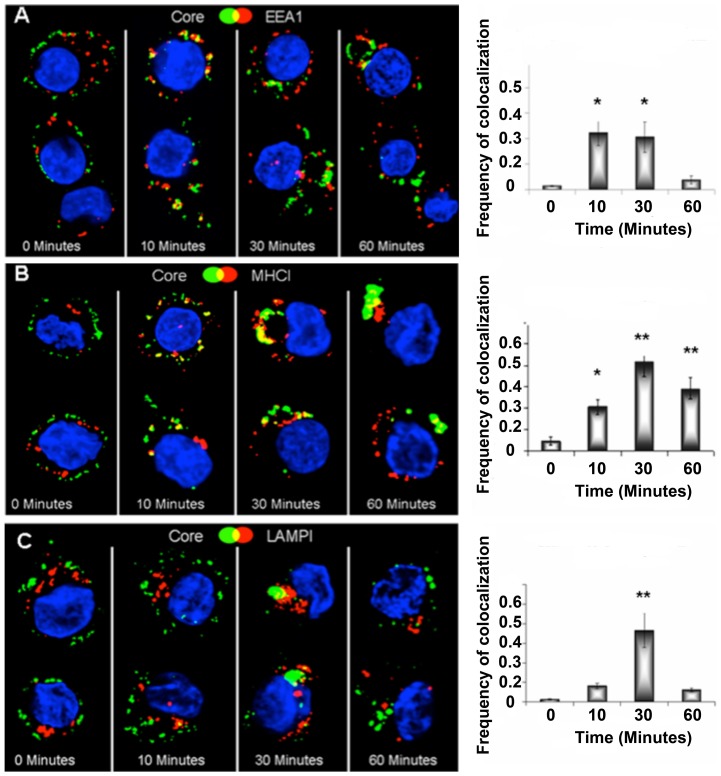
Colocalisation of M1:HBcAg and Endocytic markers. Day 6 immature DCs were incubated with M1:HBcAg on ice to allow binding and the HBcAg was fluorescently labelled. The cells were subsequently incubated at 37°C to allow internalisation. At the indicated timepoints, cells were harvested, fixed, permeabilised and stained for endocytic markers (in green) for analysis by confocal microscopy. M1:HBcAg colocalised with the early endosome marker EEA1 within 10 minutes and the late endosomal marker LAMP1 from 30 minutes. Following internalisation, M1:HBcAg was shown to colocalise with MHC I. The frequency of pixel colocalisation (on the left) was determined by Manders coefficient from 4–8 cells per treatment. Statistical significance was determined by the unpaired student *t*-test; *p<0.05, **p<0.005.

Cross presentation of exogenous antigens via the vacuolar pathway requires their internalization into late endosomes where they are processed by cathepsins and the resulting peptides loaded directly into MHC I for presentation at the cell surface (reviewed in [14]). Therefore we determined whether HBcAg targeted to MHC II on the surface of DCs could be delivered to an endosomal compartment where antigen could be degraded and presented to MHC I. To track the internalisation of M1:HBcAg by confocal microscopy DCs were incubated with particles for 30 minutes on ice to allow binding to surface MHC II followed by addition of anti-HBcAg and finally FITC-labelled secondary (mouse) antibodies. The cells were then warmed to 37°C to permit the synchronous endocytosis of labelled HBcAg and after various periods cells were fixed and analyzed by confocal microscopy. (Figure 3). HBcAg colocalised with EEA1 a marker of early endosomes within 10 minutes of warming. At later time points a significant pool of HBcAg colocalized with the late endosome marker Lamp-1 indicating progression through the endosomal pathway. It has been reported that MHC I can be internalised through early and late endosomes within which loading of peptides derived from exogenous antigens can occur prior to trafficking of the MHC I-peptide complex back to the plasma membrane [29]–[31]. Extensive co-localization of M1:HBcAgs with MHC I was evident after 30 minutes and remained significant at 60 minutes. These experiments indicate that M1 coupling can target a VLP to the surface of APCs by binding to MHC II and facilitates entry of the particle into an endosomal/lysosomal pathway. Co-localization of the HBcAg and MHC I within the same vesicular compartment suggests that degradation of the endogenous antigen by endosomal proteases could facilitate peptide loading of MHC I and thus cross presentation of exogenous antigens at the cell surface.

### M1 enhances cross presentation of HBcAg-derived epitopes to CD8^+^ T cells

**Figure 4:**
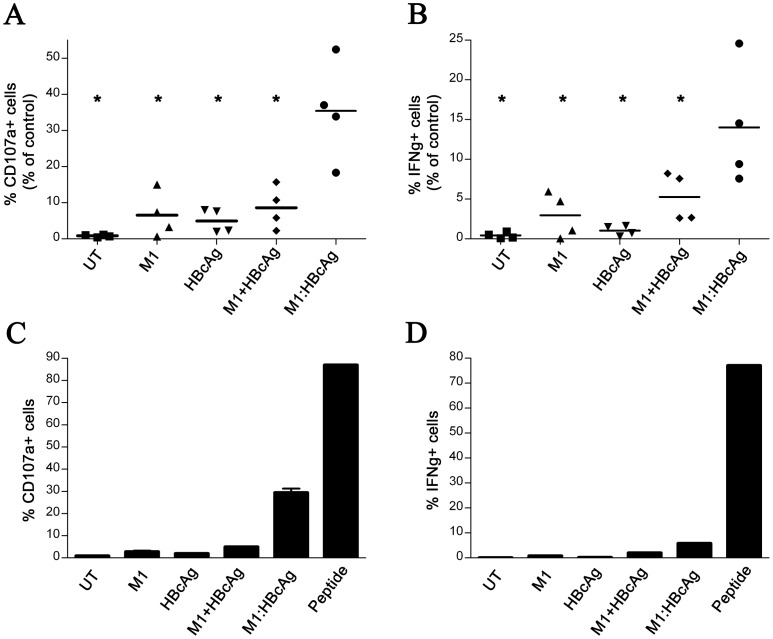
M1:HBcAg is presented on MHCI and activates HBcAg-specific CD8+ T cells. Immature DCs were pulsed with M1:HBcAg or the control antigens for 60 minutes at room temperature. Following extensive washing, the cells were incubated overnight at 37°C. Antigen-loaded cells or untreated cells (UT) were incubated with HBc_18–27_ cells for 6 hours in the presence of Brefeldin A and monensin. CD107a (A&C) expression and IFNγ (B&D) production were measured in parallel. Results in A and B indicate the T cell activation as a percentage of the positive control of DCs loaded with the molar equivalent of C_18–27_ peptide. Data are representative of 4 individual experiments. M1:HBcAg treatment resulted in significantly more T cell clones expressing CD107a and IFNγ than the control antigens. *Denotes statistical significance determined by student *t*-test, <0.05. A representative data set from one individual is presented in C & D showing mean and standard deviation of duplicate samples.

To determine whether M1 coupling to HBcAg could enhance cross presentation we examined whether DCs pulsed with VLPs could activate a human CD8^+^ T cell clone that recognizes residues 18–27 from HBcAg (HBc_18–27_) in complex with HLA-A2 MHC class I [22]. T cell activation was measured by IFNγ secretion and CD107a expression at the cell surface, a marker of CTL degranulation. Monocyte-derived DCs generated from healthy HLA-A2-positive donors were pulsed with either M1:HBcAg or unmodified HBcAg for 1 hour together with additional controls. Following overnight incubation, HBcAg-pulsed DCs were incubated with HBc_18–27_ T cells for 6 hours in the presence of monensin and BFA. The frequency of T cells positive for IFNγ and CD107a were then analysed by flow cytometry (Figure 4). As a positive control, DCs were loaded exogenously with molar equivalents of C_18–27_ peptide to reveal the maximum activation of the HBc_18–27_ T cells. M1:HBcAg-pulsed DCs induced a significantly greater number of HBc_18–27_ T cells than HBcAg alone alone or M1+HBcAg controls (i.e. non coupled). Results from an individual PBMC donor are shown in Figure 4A and B and from 4 donors in Figure 4C and D.

### Conjugation of M1 to antigen can stimulate helper T cell proliferation to augment CTL activation

**Figure 5:**
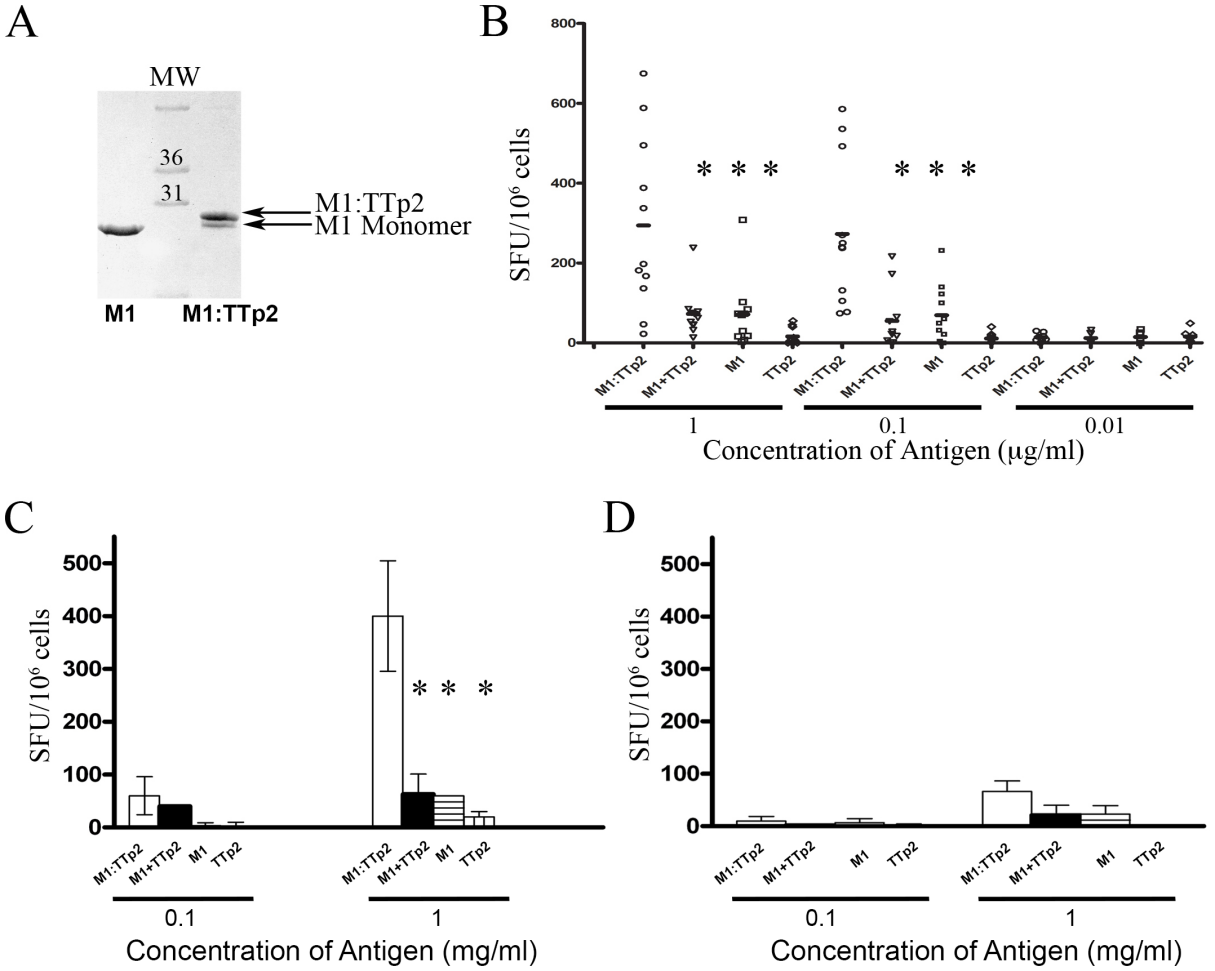
M1-conjugation improves CD4^+^ responses to the universal T_H_-epitope TTp2. TTp2 was coupled to M1 under oxidising conditions and coupling confirmed by SDS-PAGE (A). PBMC were treated with M1:TTp2 or molar equivalents of M1, TTp2 or mixed M1 and TTp2 in an IFNγ Elispot Assay (BD Biosciences). Responses from 10 individuals are shown (B). Statistically significant differences in the response observed for M1-coupled peptide and controls are denoted by * (determined by t Test). Activation of purified CD4^+^ cells (C) in the presence of the indicated antigen was assessed by Elispot assay and compared to PBMC depleted of CD4 cells in (D). Data representative of one individual's response is given showing the mean nd standard deviation of duplicate samples. Statistical significance is indicated by * where p<0.05; statistical significance was determined by the student t-test.

The activity of CD4^+^ helper T cells is critical in stimulating and shaping the CD8^+^ T cell response *in vivo*. We determined the ability of the M1 to modulate the reactivity of a well charactersised universal CD4^+^ T cell epitope, TTp2, from tetanus toxoid in human PBMC. TTp2 peptide was covalently coupled to M1 via a disulphide bond, purified by gel filtration chromatography and analysed by SDS-PAGE (Figure 5A). Coupling efficiency of 70% was routinely achieved.

The response to M1:TTp2 in PBMC isolated from these individuals was analysed by IFNγ ELISPOT assay. The M1:cTTp2 conjugate exhibited an enhanced ability to induce IFNγ secretion in all individuals compared to a molar equivalent of unmodified TTp2 peptide or M1 alone. There was a significant difference in the increase in T-cell reactivity when TTp2 was directly coupled to rather than added together with M1 The response observed to M1:cTTp2 was shown to be statistically significant relative to the controls at concentrations above 100 ng/ml (indicated by the asterisks in Figure 5B).

To confirm that the response seen in the ELISPOT assays was due to activation of CD4^+^ cells, PBMCs were depleted of CD4^+^ cells prior to ELISPOT analysis. The response to M1:cTTp2 in the CD4^+^-depleted fraction was compared to CD4^+^ cells incubated with autologous irradiated PBMC as APCs. As shown in Figure 5C, the CD4^+^ cellular fraction produced IFNγ in response to M1:cTTp2, however, the CD4^−^ cellular fraction produced minimal IFNγ (Figure 5D).

## Discussion

Virus-like particles (VLPs) are nanoscale protein assemblies that exhibit the same regular arrangment of antigenic protein seen in the native virion. This repetitive display of epitopes on the particle surface makes VLPs potent B cell antigens through efficient cross-linking of membrane-associated immunoglobulin molecules that constitute the B-cell receptor [32]. The HBV core antigen (HBcAg) is a prototypical VLP produced in the early 1980's and later developed as a particulate scaffold for incorporation of foreign peptide epitopes [33]–[35]. HBcAg-specific T cells are associated with clearance of acute and chronic hepatitis B infections [36] and the induction of HBcAg-specific CTL by prophylactic and therapeutic vaccines has proved efficacious in some animal models of infection [37]. In a recent study, immunization of HBV transgenic mice with HBcAg-pulsed DC induced HBcAg- and surface antigen (HBsAg)-specific, IFNγ-producing CD8^+^ CTL in the liver further underscoring the potential utility of VLPs as a therapeutic vaccine [38].

Immunotherapy has been proposed as an effective treatment for HBV chronic infection based on results from the adoptive transfer of HBV-specific T cells via bone marrow transplantation to HBsAg-positive patients [23], [39]. Resolution of HBV infection in these patients was associated with the activity of donor-derived HBcAg-specific CTL and CD4+ T cells [23]. Importantly, HBcAg-specific T cell activity was reported to be several fold higher compared to the T cell activity directed against HBsAg [23]. The activation of a robust CD4+ response to support effective CTL function in this study led the authors to propose that HBcAg should be included in approaches designed for HBV immunotherapy. The importance of a vigorous CD4+ T cell response is further supported by results from a trial of the CY1899 vaccine that contained the HLA A2-restricted epitope from HBcAg (HBcAg18–27). Although weak HBV-specific CTL activity was reported, this not was associated with resolution of HBV infection [40].

In comparison to live vaccines that deliver antigens to the endogenous pathway for presentation on Class I MHC, the level of HBcAg-specific CTL induced by vaccination with is relatively weak unless high doses of antigen and/or adjuvants capable of licensing APCs are employed [41], [42]. An alternative strategy to increase production of VLP-specific CTL is to target particles to a pathway of cross presentation within APCs by specific modification of the antigen. Various approaches have succeeded in improving the T cell reactivity of antigens by targeting receptors on the surface of APCs through conjugation to receptor-specific antibodies. For example, targeting of different antigens to DEC-205, an endocytic receptor on the surface of several APC subsets leads to increased T cell responses in mice and in human cells [1], [3], [43], [44]. These studies have focussed primarily on the induction of CD4^+^ T cells responses. Evidence for improved cross presentation to CD8^+^ cells has been observed in some studies though may be antigen-specific [1].

MHC class II is present on professional antigen presenting cells including DCs and thus represents a potential receptor for targeted delivery of antigen. MHC class II has been targeted using an engineered anti-MHC class II antibody containing T_H_ epitopes (so-called Troy-bodies) [45]–[47]. A novel method for targeting antigen to APCs via MHC II was reported by Dickgreber et al, who conjugated the model antigen ovalbumin (OVA) to M1, enabling specific targeting of OVA to MHC II^+^ APCs *in* vivo resulting in increased CD4^+^ and CD8^+^ OVA-specific T cells [19]. In this study we investigated whether the same approach could enhance the delivery and immunoreactivity of HBcAg VLPs and a universal helper epitope in human cells.

HBcAg conjugated to M1 were morphologically indistinguishable from unmodified HBcAg but exhibited dose-dependant and saturable binding to LG-2 cells confirming the accessibility of MHC II binding domain of M1 on the surface of the particles (Figure 2). The reduced binding of M1:HBcAg to human DCs in comparison to LG-2 was due to a reduced level of MHC-II expression at the cell surface (data not shown). In contrast, unmodified HBcAg did not bind to either cell. Previous studies demonstrated that HBcAg can bind to surface-expressed heparin sulphate on APCs via the arginine rich region spanning amino acids 145–183 and removal of this region abrogates binding to various cell lines [48], [49]. Since the VLP used in these experiments comprises only residues 1–140 of HBcAg it was not expected to bind to DCs or B cells.

To examine whether the increased affinity of M1-modified HBcAg for human DCs led to increased cross presentation, DCs were pulsed with equimolar quantities of M1-modified or native HBcAg VLPs co-cultured with a HBc_18–27_ -specific CD8 T-cell clone were generated from resolved HLA-A2^+^ HBV patient [22]. These experiments revealed a 6 fold increase in the activation of T cells measured by surface expression of the degranulation marker CD107a (Figure 4). M1 conjugation also resulted in a similar relative increase in IFNγ-producing T cells although the absolute number of IFNγ^+^ T cells was less than the number of CD107a^+^ cells possibly indicative of the need for a greater level of surface MHC I-peptide complex in the production of IFNγ following stimulation.

The cellular pathway by which exogenous particulate antigens are presented on Class I MHC remains controversial. Evidence has been presented for mechanisms that employ elements of the endogenous MHC class I-restricted presentation pathway for cross-presentation, particularly the cytosolic proteosome and transporter associated with antigen presentation (TAP) in the ER membrane [50], [51]. On the other hand, some particulate antigens appear to be processed within an endosomal compartment where epitopes can be loaded directly onto MHC I without a requirement of the proteosome or TAP function [11]. We examined the synchronous uptake of M1:HBcAgs using confocal microscopy to determine whether the antigen entered an endocytic pathway. These experiments reveal that the VLPs are rapidly taken up into EEA1^+^ early endosomes and delivered to LAMP1^+^ late endosomes within 30 minutes. Notably the frequency of co-localisation between LAMP1 and the antigen in this compartment was shared by MHCI. These experiments do not formally dissect the mechanism of cross presentation of M1:HbcAg by human DCs but confirm that the particles are efficiently endocytosed after binding to MHC II and transferred to MHC I^+^ endosomes where direct loading of epitopes within the VLP could occur. At least one other VLP has been reported to utilise a TAP-independent mechanism of cross presentation in which MHC I molecules that recycle from the cell surface can acquire epitopes derived from the VLP within an endosomal compartment [52].

Finally, as the induction of multifunctional CTL *in vivo* requires coordinate activation of CD4^+^ T helper cells, we examined whether M1 could increase the reactivity of TTp2, a universal helper epitope widely recognized by humans. TTp2 coupled to M1 via a disulphide bond induced ∼10 fold greater activation of T cells than the peptide epitope alone (Figure 5). Significantly, M1 did not increase the reactivity of either CD4^+^ or CD8^+^ T cell epitopes unless covalently coupled to the respective antigen indicating that the adjuvant effect of M1 derives from its ability to target antigens to MHC II-bearing APCs rather than from any residual mitogenic effect. It is reasonable to speculate that the ability of M1 to target antigens to M1 *in vivo* would lead to a much greater enhancement of T cell reactivity than observed in the *in vitro* comparisons reported here where the concentrations of antigens and APCs are artificially high. Trials of M1-conjugated vaccines in human subjects now have appeal to determine whether increased T cell responses to recombinant and synthetic vaccines are observed *in vivo* to realise the therapeutic potential.
